# Loop-Mediated Isothermal Amplification Coupled With Nanoparticle-Based Lateral Biosensor for Rapid, Sensitive, and Specific Detection of *Bordetella pertussis*

**DOI:** 10.3389/fbioe.2021.797957

**Published:** 2022-02-08

**Authors:** Chunrong Sun, Fei Xiao, Jin Fu, Xiaolan Huang, Nan Jia, Zheng Xu, Yi Wang, Xiaodai Cui

**Affiliations:** Experiment Center, Captital Institute of Pediatrics, Beijing, China

**Keywords:** Bordetella pertussis, LAMP, lateral flow biosensor, rapid diagnosis, qPCR

## Abstract

*Bordetella pertussis* is the most frequent causative agent for pertussis, which is a highly contagious disease. Here, we developed a method based on loop-mediated isothermal amplification (LAMP) and nanoparticle-based lateral flow biosensor (LFB) for the timely diagnosis of *B. pertussis* infections. A set of six primers was designed for LAMP reactions, and the LAMP results were rapidly and visually indicated using LFB. The recommended condition for the *B. pertussis* LAMP reactions is 40 min at 66°C. Our results confirmed that the LAMP-LFB assay could specifically detect *B. pertussis* and did not cross-react with non-*B. pertussis* isolates. The sensitivity of the *B. pertussis* LAMP-LFB assay was 50 fg per reaction. In particular, 108 nasopharyngeal swab (NPS) samples were collected to evaluate the *B. pertussis* LAMP-LFB assay, and the results were compared with those of the quantitative PCR (qPCR) method. The positive rates of *B. pertussis* LAMP-LFB and qPCR were 40.7% and 38.8%, respectively, and the agreement between the LAMP-LFB and qPCR results was 98%, with a kappa value of 0.96. The whole process of LAMP-LFB can be completed within 1 h, which is much shorter than that of qPCR, including about 15 min of rapid DNA extraction, 40 min of LAMP reaction, and within 2 min of the LFB test. Collectively, the *B. pertussis* LAMP-LFB assay developed in this report offers a new option for the rapid, reliable, and simple diagnosis of *B. pertussis* infections.

## Introduction

*B*ordetella *pertussis* mainly causes pertussis, a highly infectious, even fatal illness in children. In the past few years, the resurgence of pertussis has become a global public health issue in spite of high vaccination rates (Wood and McIntyre, 2008; Cherry, 2013; Mooi et al., 2014; Yeung et al., 2017; Del Valle-Mendoza et al., 2021; Pandolfi et al., 2021). In China, *B. pertussis* infections are becoming more and more prevalent even with over 99% vaccination coverage in children during the last 20 years (Liu et al., 2018; Fu et al., 2019; Zhang et al., 2019; Kang et al., 2022). Consequently, a rapid and reliable laboratory diagnosis of *B. pertussis* is particularly important (Cherry et al., 2005; de Greeff et al., 2010; Tao et al., 2019; Wu et al., 2019; Macina and Evans, 2021)*.*

The current approaches to the diagnosis of pertussis include direct fluorescent antibody (DFA) assay, culture-based approaches, serodiagnosis, and PCR assays (van der Zee et al., 2015). DFA is a simple fluorescent antibody examination done through microscopic observation directed to the pathogen*,* but lacks both specificity and sensitivity (Chia et al., 2004; van der Zee et al., 2015). Culture is the gold standard diagnostic test, but with very low sensitivity. Meanwhile, the process of culture is laborious and time-consuming, which do not help with timely treatment, especially for infants too young to be vaccinated. Serodiagnosis is another technique earlier used for confirmation of the clinical diagnosis of pertussis, but it suffers persistent problems, including cross-reactivity with other bacteria, not only with *Bordetella* species, and the interference of previous vaccination or previous infections (Chia et al., 2004; Mertens et al., 2007; Bock et al., 2012). At present, PCR-based assays [e.g., conventional PCR, real-time PCR (RT-PCR), and quantitative PCR (qPCR)] have been established for the detection of *B. pertussis* (Roorda et al., 2011; Tatti et al., 2011; Abu Raya et al., 2012; Gao et al., 2014; Pittet et al., 2014). In particular, RT-PCR and qPCR use labeled probes to release a reporter or high-resolution melt (HRM) analysis to the amplicon, thus allowing the real-time monitoring of the amplification results. However, RT-PCR and qPCR examination is rarely available in primary medical institutions or in underdeveloped areas due to the high requirements of equipment and skilled professionals for a PCR laboratory.

Loop-mediated isothermal amplification (LAMP) is a newly developed amplification technique amplifying DNA at an isothermal condition, which can be satisfied merely by a water bath or a heater. By using six primers directing the different regions of the target sequence, this method showed high specificity, sensitivity, and efficiency (Notomi et al., 2000; Kamachi et al., 2006; Fujino et al., 2015; Notomi et al., 2015). In this report, we employed LAMP to amplify the target sequence of the pertussis toxin (PT) promoter, *ptxA* (pertussis toxin subunit 1), assumed to be specific for *B. pertussis* (Grimprel et al., 1993; Nygren et al., 2000). The LAMP products were judged using nanoparticle-based lateral flow biosensor (LFB), a method for the detection of nucleic acid and protein molecules (Huang et al., 2021; Huang et al., 2020; Huang et al., 2019), which can visually, rapidly and objectively indicate the results without the need for any extra instrument. The *B. pertussis* LAMP-LFB assay was further evaluated by applying it to clinical nasopharyngeal swab (NPS) samples.

## Materials and Methods

### Reagents and Instruments

The DNA isothermal amplification kit, visual detection reagent (VDR), and the nanoparticle-based LFB were obtained from Huidexin Biotech Co., Ltd. (Tianjin, China). The primers and labeled primers used in this study were synthesized by AoKe Biotech (Beijing) Co., Ltd. (Beijing, China). The *B. pertussis* isolate and qPCR kits were purchased from Beijing Transgen Biotech Co., Ltd. (Beijing, China) and Shanghai ZJ Bio-Tech Co., Ltd. (Shanghai, China). Real-time turbidimeter LA-320C was purchased from Eiken Chemical Co., Ltd. (Tokyo, Japan).

### Primer Design

**FIGURE 1:**
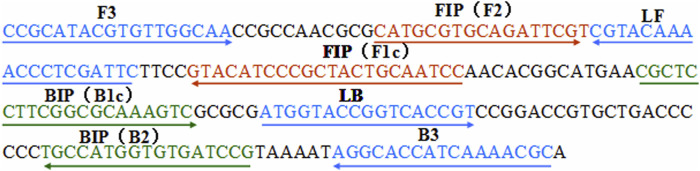
Locations of the primer sequences used in this study for targeting the pertussis toxin (PT) promoter region of *Bordetella pertussi*s. *Right* and *left arrows* show the sense and complementary sequences, respectively. The *colored text* indicates the positions of the primers, including two outer primers (F3 and B3), two inner primers (FIP and BIP), and two loop primers (LF* and LB).

A set of six primers, including two outer primers (F3 and B3), two inner primers (FIP and BIP), and two loop primers (LF* and LB), was designed based on the specific pertussis toxin (PT) promoter gene of *Bordetella pertussis* (genome positions 159549–159755; GeneBank: BX640422) using Primer Premier 5.0. The sequences, locations, and modifications of the primers used in this report are shown in Figure 1 and Table 1.

**TABLE 1:** Sequences of the primers used in this study

Assay	Primers	Sequence (5’–3’)
LAMP	BT-F3	CCGCATACGTGTTGGCA
	BT-B3	GCGTTTTGATGGTGCCT
	BT-FIP	GGA​TTG​CAG​TAG​CGG​GAT​GTA​C-CAT​GCG​TGC​AGA​TTC​GT
	BT-BIP	CGC​TCC​TTC​GGC​GCA​AAG​TC-CGG​ATC​ACA​CCA​TGG​CA
	BT-LF	GAA​TCG​AGG​GTT​TTG​TAC​G
	BT-LB	ATGGTACCGGTCACCGT
Labeled primer^a^	BT-LF*	5’-Fam-GAATCGAGGGTTTTGTACG-3’

a Fam, carboxyfluorescein. BT-LF*, 5’ labeled with Fam used in the loop-mediated isothermal amplification/nanoparticle-based lateral flow biosensor (LAMP-LFB) assay

### LAMP Reaction

LAMP reactions were performed as a one-step reaction in a 25-μl mixture containing 12.5 μl reaction buffer, 0.1 μmol L^−1^ each of the displacement primers (F3 and B3), 0.4 μmol L^−1^ each of the inner primers (FIP and BIP), 0.2 μmol L^−1^ each of the loop primers (LF* and LB), 1.0 μl *Bst* DNA polymerase (8 U), 0.5 μl biotin-14-dCTP (Huidexin Biotech Co., Ltd., Tianjin, China), 1.0 μl VDR, and 1.0 μl template for pure culture (5 µl for clinical sample).

### Lateral Flow Biosensor (LFB) Test

LFB was constructed according to the previous report (Li et al., 2020). Briefly, LFB contained a sample pad, a conjugate pad, a nitrocellulose(NC)membrane (#Whatma99; Jie-Yi biotech Co., Ltd, Shanghai, China) and a absorbent pad (Huidexin Biotech Co., Ltd, Tianjin, China). On the conjugated pad, the detector reagents (dye streptavidin-coated gold nanoparticles (streptavidin-GNPs)) were laminated. As for the control line (CL) and test line (TL), Biotin-BSA and anti-FAM were immobilized on the NC membrane, respectively. The finally assembled biosensors were packaged in plastic box and conserved with silica gel desiccant at room temperature. For indicating the LAMP results, a 5 µl aliquot of LAMP reaction products was added to the sample pad, followed with 100 µl running buffer (10 mM PBS, PH 7.4 with 1% Tween 20), The results was indicated within 2 min, two red lines at TL and CL represent positive and one red line at CL means negative.

### Optimal Temperature for the *B. pertussis* LAMP Assay

The amplification temperatures were optimized from 60°C to 67°C with 1°C intervals for the optimal temperature of the LAMP reaction. The DNA template of *B. pertussis* was used as a positive control and distilled water (DW) was used as the blank control. The LAMP reactions were monitored using real-time turbidity measurements.

### Specificity of the *B. pertussis* LAMP-LFB Assay

To evaluate the specificity of the *B. pertussis* LAMP-LFB assay, the DNA templates from *B. pertussis* and non-*B. pertussis* strains (Table 2) were tested at least twice with the assay*.*

**TABLE 2:** Bacterial strains used to determine the specificity of loop-mediated isothermal amplification (LAMP)

Bacteria	Strain no. (source of strains)	No. of strains	*B. pertussis* LAMP-LFB
*Bordetella pertussis*	Isolated strains (CIP)	3	P
Enteroinvasive *Escherichia coli*	Isolated strains (CDC)	1	N
Enteroadherent *Escherichia coli*	Isolated strains (CDC)	1	N
Enterotoxic *Escherichia coli*	Isolated strains (CDC)	1	N
Enteropathogenic *Escherichia coli*	Isolated strains (CDC)	1	N
Shiga toxin-producing *Escherichia coli*	Isolated strains (CDC)	1	N
*Streptococcus suis*	Isolated strains (CDC)	2	N
*Citrobacter*	Isolated strains (CDC)	2	N
*Listeria innocua*	Isolated strains (CDC)	1	N
*Listeria monocytogenes*	Isolated strains (CDC)	1	N
*Listeria ivanovii*	Isolated strains (CDC)	1	N
*Klebsiella pneumoniae*	Isolated strains (CDC)	3	N
*Streptococcus salivarius*	Isolated strains (CDC)	1	N
*Mycobacterium tuberculosis*	Isolated strains (CDC)	1	N
*Corynebacterium striatum*	Isolated strains (CDC)	1	N
*Nocardia asteroides*	Isolated strains (CDC)	1	N
*Moraxella catarrhalis*	Isolated strains (CDC)	1	N
*Stenotrophomonas maltophilia*	Isolated strains (CDC)	1	N
*Staphylococcus epidermidis*	Isolated strains (CDC)	1	N
*Staphylococcus amber*	Isolated strains (CDC)	1	N
*Staphylococcus haemolyticus*	Isolated strains (CDC)	1	N
N.Lac	Isolated strains (CDC)	1	N
*Neisseria meningitidis*	Isolated strains (CDC)	1	N
*Streptococcus pneumoniae*	Isolated strains (CDC)	1	N
*Streptococcus pyogenes*	Isolated strains (CDC)	1	N
*Pseudomonas aeruginosa*	Isolated strains (CDC)	4	N
*Monilia albicans*	Isolated strains (CDC)	2	N
*Bacillus cereus*	Isolated strains (CDC)	1	N
*Streptococcus aureus*	Isolated strains (CDC)	1	N
*Salmonella*	Isolated strains (CDC)	2	N
*Shigella sonnei*	Isolated strains (CDC)	1	N
*Shigella baumannii*	Isolated strains (CDC)	1	N
*Enterococcus faecalis*	Isolated strains (CDC)	2	N

Only *Bordetella pertussis* strains were detected as positive, indicating the high specificity of the *B. pertussis* loop-mediated isothermal amplification/nanoparticle-based lateral flow biosensor (LAMP-LFB) assay.

*CIP*, Capital Institute of Pediatrics; *CDC*, Chinese Center for Disease Control and Prevention.; *P*, positive; *N*, negative

### Sensitivity of the *B. pertussis* LAMP-LFB Assay

To verify the limit of detection (LoD), the DNA templates of *B. pertussis* were serially diluted (5 ng ml^−1^; 500, 50, and 5 pg ml^−1^; and 500, 50, and 5 fg ml^−1^) for the LAMP assay, and 1 μl of each serial dilution or DW was added to the reaction mixtures. The LoD of the *B. pertussis* LAMP assay was determined using real-time turbidity measurement, VDR, and the LFB test. All tests were repeated at least twice.

### Optimal Amplification Time for the *B. pertussis* LAMP Assay

Serially diluted templates were applied to obtain the optimal amplification time. LAMP reactions were conducted at 66°C with reaction times ranging from 10 to 40 min, with 10-min intervals. Each reaction time was verified twice.

### Application of the *B. pertussis* LAMP-LFB Assay in Clinical Specimens

A total of 108 NPS samples collected from patients suspected of pertussis in the clinics of the Children’s Hospital affiliated with the Capital Institute of Pediatrics from January 1, 2019 to December 30, 2020 were retrospectively used. All samples were obtained with informed consent signed by the guardians of the participants. Nucleic extractions from these samples were firstly used for clinical and laboratory diagnosis. A volume of 5 μl DNA template was collected from the remaining samples for the *B. pertussis* LAMP-LFB assay. The results of the *B. pertussis* LAMP-LFB assay were compared with those of the qPCR assay for identical samples. All procedures were reviewed and approved by the Ethics Committee of the Capital Institute of Pediatrics.

### Statistical Analysis

A comparison between two methods, qPCR and LAMP-LFB assay, was analyzed using the *χ* ^2^ test with SPSS software (version 11.5). A *p* < 0.05 was considered statistically significant.

## Results

### Confirming the Feasibility of the *B. pertussis* LAMP Reaction

**FIGURE 2:**
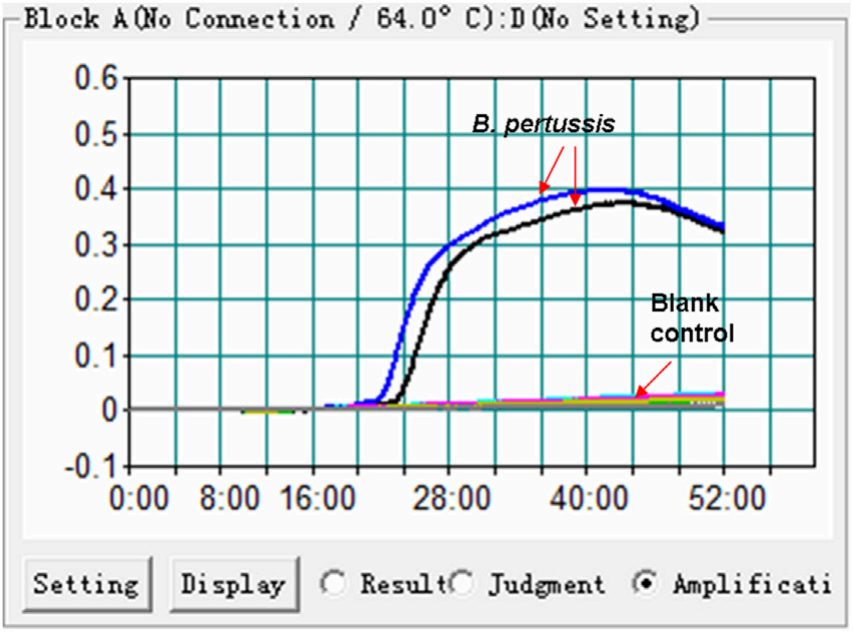
Effectiveness of the primer set for the *Bordetella pertussis* loop-mediated isothermal amplification (LAMP) reaction. The DNA templates extracted from *B. pertussis* strains were effectively amplified with LAMP reaction at 64°C, while there was no reaction for the blank controls (distilled water, DW).

The feasibility of the *B. pertussis* LAMP primer set (Figure 1 and Table. 1) was confirmed using DNA templates extracted from *B. pertussis* strains. The LAMP reaction was conducted at 64°C for ∼60°min. The results showed that the templates were effectively amplified, and no amplifications were observed for DW (blank control) (Figure 2). Thus, the primer set designed in our report was used as the candidate to establish the *B. pertussis* LAMP-LFB assay.

### Optimal Temperature for the *B. pertussis* LAMP Reaction

**FIGURE 3:**
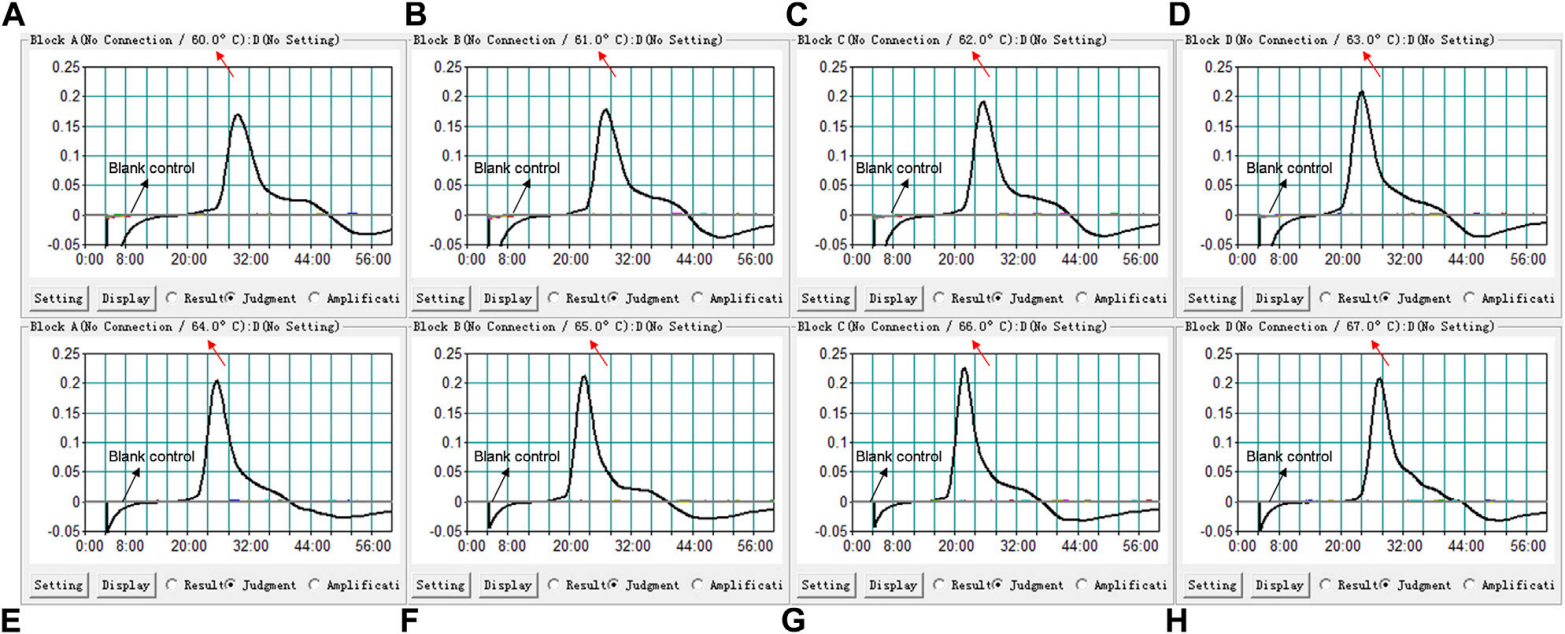
Temperature optimization for the loop-mediated isothermal amplification (LAMP) assay. LAMP reactions detecting *Bordetella pertussis* were conducted using real-time turbidimetry and kinetic curves **(A**–**H)** at different temperatures ranging from 60°C to 67°C were acquired, showing that 66°C was optimal for the *B. pertussis* LAMP reaction.

We used eight different temperatures ranging from 60°C to 67°C at 1°C intervals for 40°min to conduct the *B. pertussis* LAMP reaction for the optimal temperature. As shown in Figure 3, faster amplification was observed at 66°C, which was subsequently used for the *B. pertussis* LAMP-LFB reaction as the optimal temperature in this report.

### Sensitivity of the LAMP-LFB Assay for the Detection of *B. pertussis*

**FIGURE 4:**
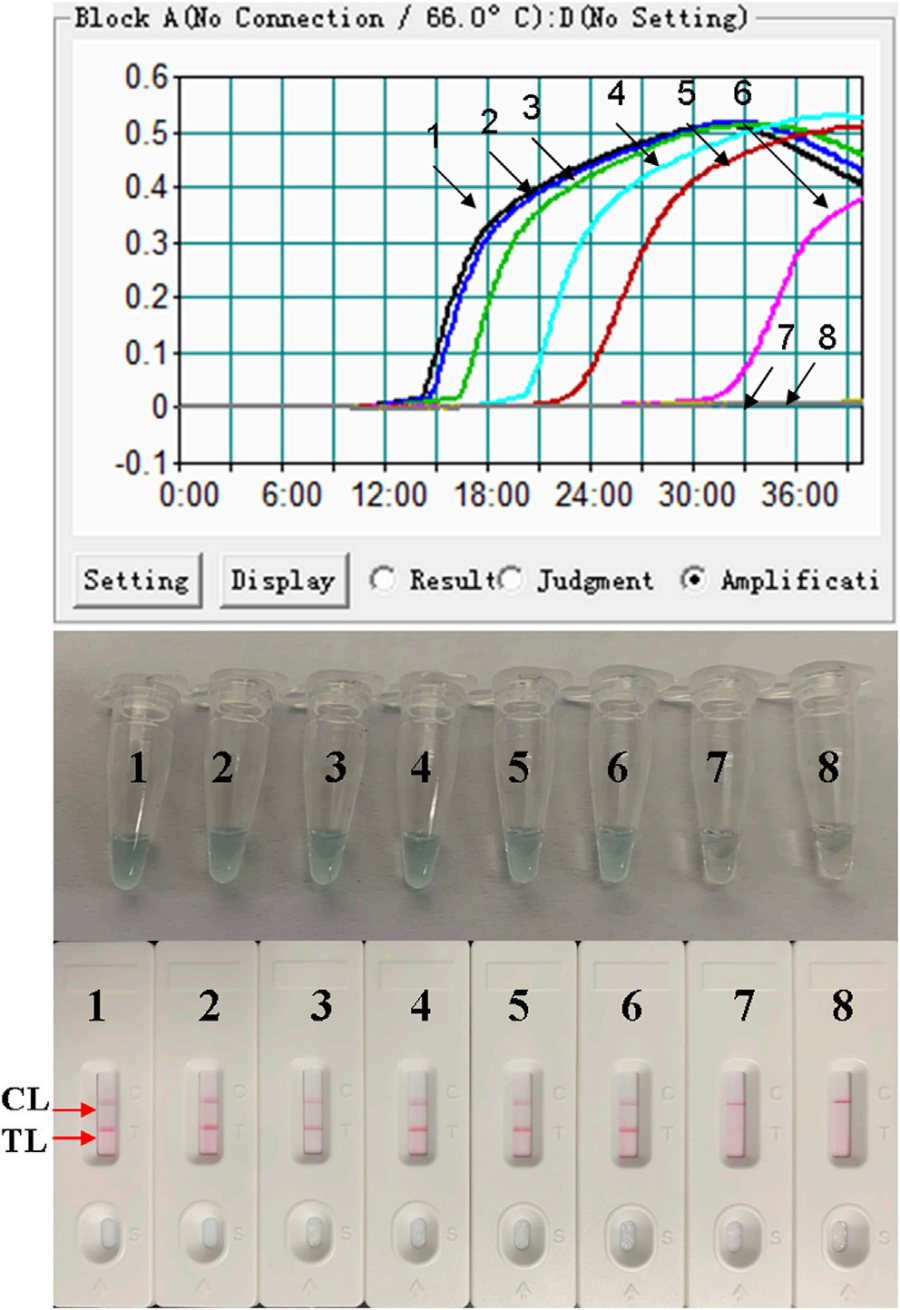
Sensitivity confirmation of the *Bordetella pertussis* loop-mediated isothermal amplification (LAMP) assay. The sensitivity of the assay was analyzed using 10-fold serial dilutions from 5 ng to 5 fg per reaction. The LAMP reactions with various levels of DNA templates were recorded with turbidity curves, and the products were indicated by a visual detection reagent (VDR) and a nanoparticle-based lateral flow biosensor (LFB). Turbidity curves/tubes/strips *1*–*7* represent different concentrations of DNA: 5 ng; 500, 50, and 5 pg; and 500, 50, and 5 fg per reaction. Turbidity curve/tube/strip *8* represents the blank control. *TL*, test line; *CL*, control line.

The DNA templates of *B. pertussis* were serially diluted to examine the LoD of the *B. pertussis* LAMP-LFB assay. The results were indicated by LFB and further confirmed by turbidity and VDR. As shown in Figure 4, the LoD of the *B. pertussis* LAMP-LFB assay was as low as 50 fg (∼12 copies) per reaction.

### Optimal Time for the *B. pertussis* LAMP Reaction

**FIGURE 5:**
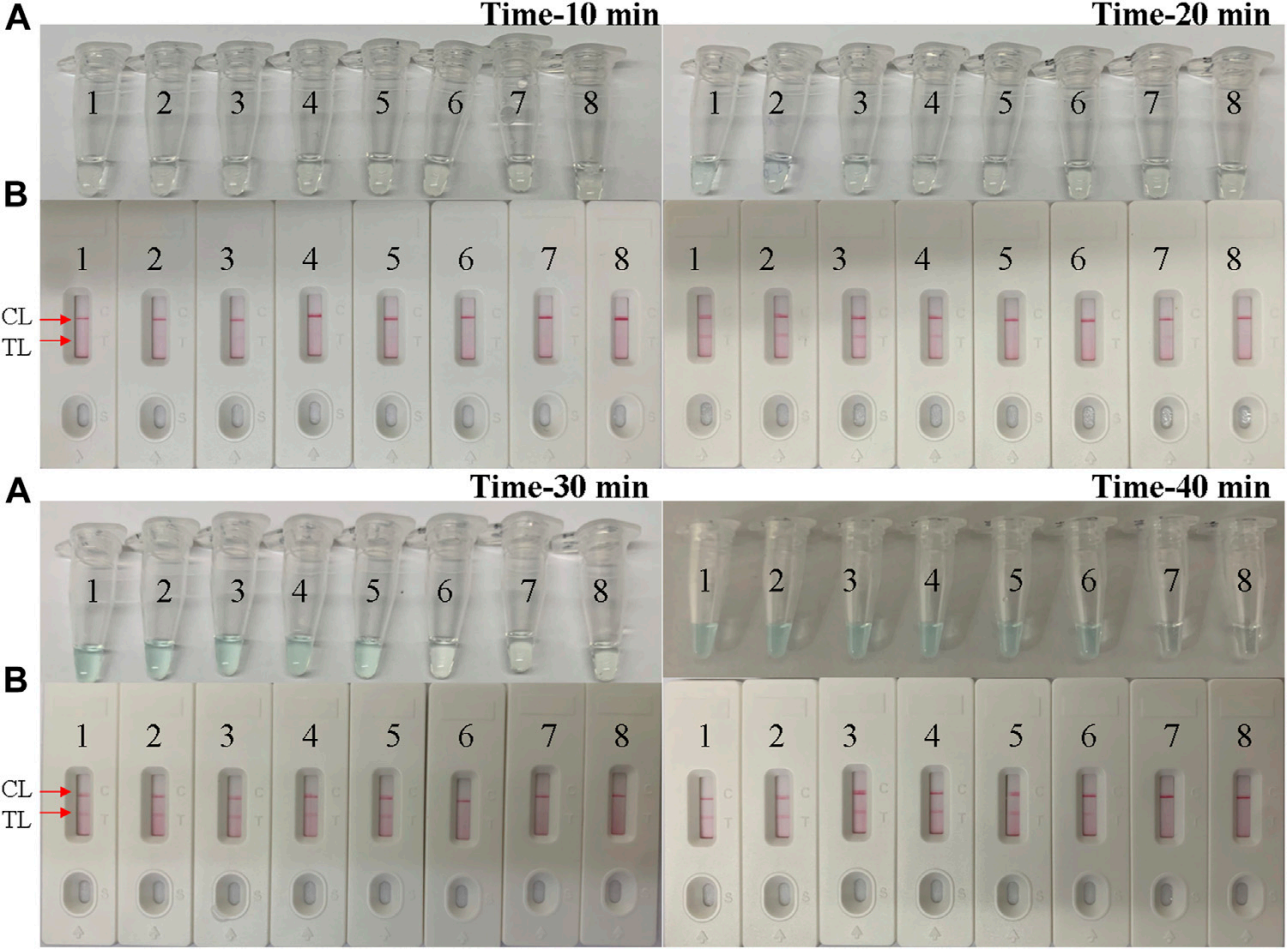
Optimal time for the *Bordetella pertussis* loop-mediated isothermal amplification (LAMP) assay. Tenfold serial dilutions of the *B. pertussis* templates were used for the optimization of time. The LAMP reactions were conducted at different times from 10 to 40 min, with 10-min intervals. The products were indicated by the visual detection reagent **(A)** and a nanoparticle-based lateral flow biosensor (LFB) test **(B)**. Tubes/strips *1*–*7* represent serial dilutions of DNA: 5 ng; 500, 50, and 5 pg; and 500, 50, and 5 fg per reaction. Tube/strip *8* represents the blank control. *TL*, test line; *CL*, control line.

We examined a total of four reaction times, 10–40 min with 10-min intervals, for the optimal amplification time of the *B. pertussis* LAMP assay. As shown in Figure 5, at 40 min, the amplicon of the diluted template at the LoD level was successfully detected by LFB, in which two red lines appeared respectively at the location of the test line (TL) and the control line (CL) on the strips. Therefore, 40 min was subsequently used as the optimal time for the *B. pertussis* LAMP assay. Hence, the whole procedure, which included rapid DNA extraction (15 min), LAMP reaction (40 min), and LFB indication (2 min), takes approximately 60 min, which is only half of that of qPCR.

### Specificity of the *B. pertussis* LAMP-LFB Assay

**FIGURE 6:**
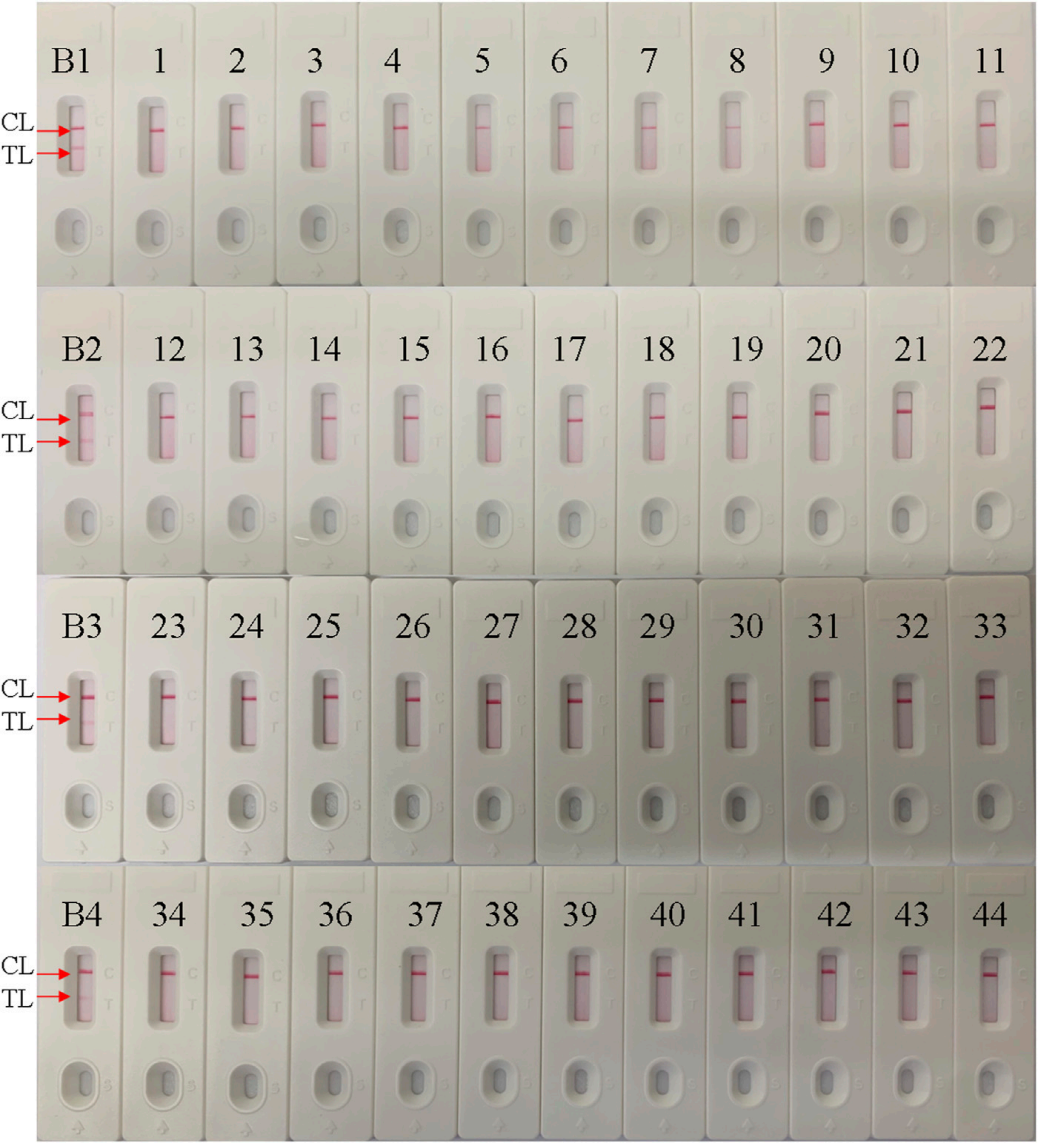
Specificity conformation for the *Bordetella pertussis* loop-mediated isothermal amplification (LAMP) assay. A lateral flow biosensor was applied for the LAMP products. Strips *B1*–*B4* represent DNA isolation of the *B. pertussis*-positive clinical samples; strips *1*–*44* represent the other bacterial strains, shown in Table 2. *TL*, test line; *CL*, control line.

The specificity of the *B. pertussis* LAMP-LFB assay was examined using *B. pertussis* and non-*B. pertussis* strains (Table 2). As in the results shown in Figure 6, only CL lines appeared on the LFB strips of the non-*B. pertussis* strains and blank controls, while two red lines appeared at the CL and TL locations on the strips of the *B. pertussis* strains, suggesting the specificity of the primers in that only DNA isolates from *B. pertussis* strains could be amplified.

### Application of the *B. pertussis* LAMP-LFB Assay in Clinical Specimens

**FIGURE 7:**
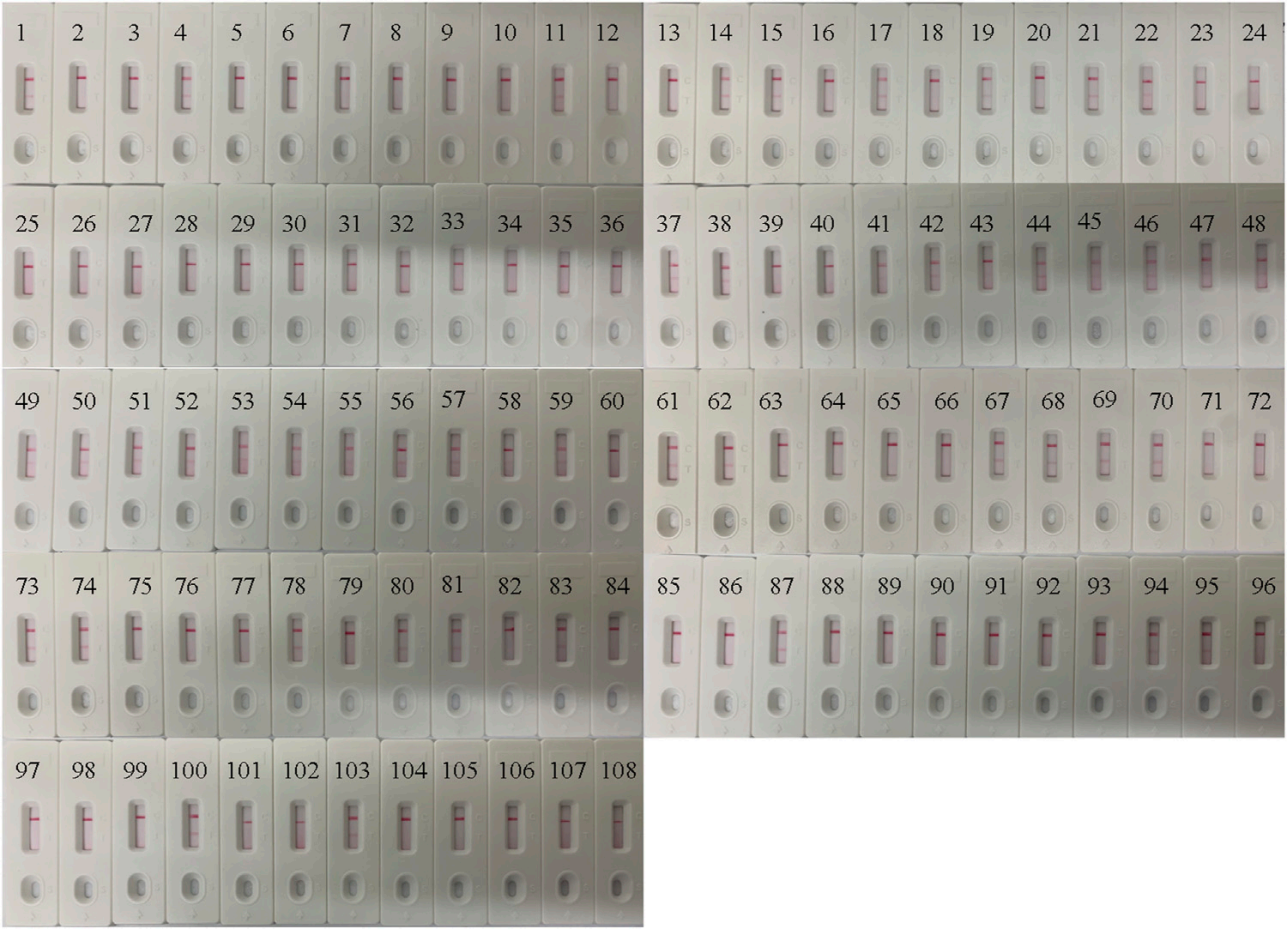
Application of the *Bordetella pertussis* loop-mediated isothermal amplification/nanoparticle-based lateral flow biosensor (LAMP-LFB) assay in clinical specimens. A total of 108 nasopharyngeal swab (NPS) samples were detected by the LAMP-LFB assay, with the results showing 44 samples testing positive.

In order to confirm its clinical application value, the optimized *B. pertussis* LAMP-LFB assay was used to detect 108 NPS samples, which were also detected using qPCR. The results (Figure 7) showed that 44 samples (40.7%) tested positive with the LAMP-LFB assay, while 42 samples (38.8%) tested positive with the qPCR. The agreement in the results between the qPCR and the LAMP-LFB assay was 98%, with a kappa value of 0.96.

## Discussion

As a previous major cause of infant death, the morbidity and mortality of *B. pertussis* infections have significantly declined, benefitting from general vaccinations in childhood since 1950s. However, in the last 20 years, global resurgence was found in several highly vaccinated populations (Mooi et al., 2014; Yeung et al., 2017; Liu et al., 2018; Fu et al., 2019; Tao et al., 2019; Wu et al., 2019; Zhang et al., 2019; Kang et al., 2022). An estimation of the infection frequency derived from seroprevalence studies among adolescents and adults revealed a high circulation rate (1%–9% annually) in vaccinated populations (Wood and McIntyre, 2008; Cherry, 2013). Thus, the early detection of *B. pertussis* enables not only timely treatment, especially for infants much more fragile to the infection, but also the prevention of transmission and unnecessary diagnostic procedure, especially during an outbreak.

In this report, a simple LAMP-LFB assay for the detection of *B. pertussis* was designed and validated by its application in clinical samples. The assay takes less time, ∼60 min, with 15 min for rapid DNA extraction, 40 min for LAMP reaction, and 2 min for LFB detection, which was more rapid than that of traditional molecule-based diagnosis (e.g., PCR-based assay). Moreover, the significant advantage of the LAMP reaction is the isothermal condition, so the assay can be easily carried out under any experiment conditions with just a thermostatic water bath or a heater. The LFB test can subjectively indicate the results of the amplicons within 2 min.

As a molecular technique, the efficiency of the LAMP reaction is mostly decided by the primers and its targeting sequence. In previous studies, the sequence of the PT promoter has been considered as the very specific region for the diagnosis of *B. pertussis*. Comparison with previously used popular targeting sequences, such as insert sequence (IS) 1002 or IS481, demonstrated that primers targeting the PT gene showed marked reliability, selectivity, and sensitivity (van der Zee et al., 2015; Kilgore et al., 2016; Wu et al., 2019). We designed six primers targeting the PT sequence for the LAMP assay. The efficiency was conformed, as shown in Figure 3G, with the amplification peaking at 20–24 min under the condition of 66°C by turbidimetry. The specificity was confirmed by using the genomic templates of other bacterial strains, shown in Table 2. The high specificity (100%) indicated that the LAMP-LFB assay we investigated was reliable for the detection of *B. pertussis*.

In addition to the specificity, sensitivity was confirmed by using serial dilutions of genomic DNA. As shown in Figure 4, the lowest detection level of the LAMP-LFB assay was 50 fg of the DNA templates isolated from pure culture of *B. pertussis*. Since the LF primers were labeled with Fam at the 5’ end and biotin-14-dCTP was used in the reaction system, the amplicons positive in LAMP were simultaneously labeled with Fam and biotin, which can be detected by LFB. Thus, the positive LAMP amplicons displayed two red lines (CL and TL), while the negative reactions and the blank control displayed only the CL line when the reaction products were tested using LFB. The use of biotin-14-dCTP in the LAMP reaction instead of a biotin-labeled primer such as FIP absolutely avoided the interference of primer dimers containing Fam-labeled LF and biotin-labeled FIP.

For evaluation of the feasibility of the LAMP-LFB assay in the clinical diagnosis of *B. pertussis* infections, we compared the test with qPCR, the established method for *B. pertussis* diagnosis. Of the 108 clinical samples tested, 42 (38.8%) were positive by qPCR and 44 (40.7%) were positive by the LAMP-LFB assay. According to manual in the kit, the LoD of the qPCR assay used in this report is 250 copies, which is equal to about 1 pg DNA template, while the *B. pertussis* LAMP-LFB assay we conducted displayed better sensitivity with an LoD of 50 fg DNA template. For this reason, the LAMP-LFB assay yielded a higher positive rate than that of the qPCR assay in the clinical samples. Besides the lower LoD of the qPCR kit, the presence of some inhibitors specific to qPCR may have also contributed to the lower positive rates of detections. Therefore the application of the *B. pertussis* LAMP-LFB assay was verified to be sensitive and specific for the clinical diagnosis of *B. pertussis* infections. Moreover, the lower cost of the LAMP-LFB assay could also benefit its extensive application prospects in resource-limited laboratories.

## Data Availability

The datasets presented in this study can be found in online repositories. The names of the repository/repositories and accession number(s) can be found in the article/supplementary material.
